# CROSS-CULTURAL ADAPTATION AND VALIDATION OF THE MONTREAL CHILDREN’S HOSPITAL FEEDING SCALE INTO BRAZILIAN PORTUGUESE

**DOI:** 10.1590/1984-0462/2021/39/2019377

**Published:** 2021-02-24

**Authors:** Patricia Barcellos Diniz, Simone Chaves Fagondes, Maria Ramsay

**Affiliations:** a Universidade Federal do Rio Grande do Sul, Porto Alegre, RS, Brasil.; b McGill University, Montreal, Quebec, Canadá.

**Keywords:** Feeding behavior, Validation study, Child, ROC curve, Comportamento alimentar, Estudos de validação, Criança, Curva ROC

## Abstract

**Objective:**

To cross-culturally adapt and validate the Montreal Children’s Hospital Feeding Scale (MCH-FS) into Brazilian Portuguese.

**Methods:**

The MCH-FS, originally validated in Canada, was validated in Brazil as *Escala Brasileira de Alimentação Infantil* (EBAI) and developed according to the following steps: translation, production of the Brazilian Portuguese version, testing of the original and the Brazilian Portuguese versions, back-translation, analysis by experts and by the developer of the original questionnaire, and application of the final version. The EBAI was applied to 242 parents/caregivers responsible for feeding children from 6 months to 6 years and 11 months of age between February and May 2018, with 174 subjects in the control group and 68 ones in the case group. The psychometric properties evaluated were validity and reliability.

**Results:**

In the case group, 79% of children were reported to have feeding difficulties, against 13% in the control group. The EBAI had good internal consistency (Cronbach’s alpha=0.79). Using the suggested cutoff point of 45, the raw score discriminated between cases and controls with a sensitivity of 79.4% and specificity of 86.8% (area under the ROC curve=0.87).

**Conclusions:**

The results obtained in the validation process of the EBAI demonstrate that the questionnaire has adequate psychometric properties and, thus, can be used to identify feeding difficulties in Brazilian children from 6 months to 6 years and 11 months of age.

## RESUMO

**Objetivo:**

Realizar a adaptação transcultural e a validação da escala *Montreal Children’s Hospital Feeding Scale* (MCH-FS) para a língua portuguesa falada no Brasil.

**Métodos:**

A MCH-FS, originalmente validada no Canadá, foi validada no Brasil como Escala Brasileira de Alimentação Infantil (EBAI) e desenvolvida a partir das seguintes etapas: tradução, montagem da versão em português brasileiro, teste da versão em inglês e da versão em português brasileiro, retrotradução, análise por *experts* e autora do questionário original e aplicação da versão final em estudo. A EBAI foi aplicada em 242 pais/cuidadores responsáveis pela alimentação de crianças de seis meses a seis anos e 11 meses de idade no período de fevereiro a maio de 2018, sendo 174 no grupo controle e 68 no grupo dos casos. As propriedades psicométricas avaliadas foram validade e confiabilidade.

**Resultados:**

No grupo dos casos, 79% dos pais/cuidadores relataram dificuldades alimentares, e no grupo controle, 13%. A EBAI apresentou boa consistência interna (alfa de Cronbach=0,79). Utilizando-se o ponto de corte sugerido de 45, o escore bruto (*raw score*) diferenciou casos de controles com sensibilidade de 79,4% e especificidade de 86,8% (área sob a curva ROC=0,87).

**Conclusões:**

Os resultados obtidos na validação da EBAI evidenciaram medidas psicométricas adequadas. Portanto, a escala pode ser utilizada na identificação de dificuldades alimentares em crianças brasileiras de seis meses a seis anos e 11 meses idade.

## INTRODUCTION

Eating difficulties can occur in 20-35% of the pediatric population, with neurotypical development. These rates can reach 80% in populations at risk, such as those with developmental delays, premature births and/or chronic and complex medical conditions.^1^ ^,^ ^2^ ^,^ ^3^ ^,^ ^4^ ^,^ ^5^ ^,^ ^6^ ^,^ ^7^ ^,^ ^8^ ^,^ ^9^ Feeding difficulties are a high-impact clinical problem, with negative consequences for the child,^3^ ^,^ ^8^ ^,^ ^9^ including failure to thrive, malnutrition, lethargy, developmental delay, aspiration, invasive medical procedures, admission to the inpatient unit and even death.^10^ Likewise, feeding difficulties significantly affect family relationships,^7^ leading to excessive stress during meals,^1^ ^,^ ^2^ ^,^ ^8^ ^,^ ^9^ ^,^ ^11^ hindering many aspects of the life and general well-being of both children and their families.

Due to the high prevalence^1^ ^,^ ^2^ ^,^ ^3^ ^,^ ^4^ ^,^ ^5^ ^,^ ^6^ ^,^ ^7^ ^,^ ^8^ ^,^ ^9^ ^,^ ^10^ ^,^ ^12^ and the negative consequences of feeding difficulties, health professionals should have access to a valid and reliable screening instrument, of clinical applicability, able to quickly identify the complaints of parents or legal guardians on their children’s feeding difficulties. Thus, referral to specialists can be carried out as soon as the problem is identified.^12^ ^,^ ^13^ However, to date, few self-completed instruments, applicable to parents/caregivers, with standardized psychometric measures, have been validated to reliably identify their perceptions of children’s feeding difficulties.^3^ ^,^ ^10^ ^,^ ^14^ ^,^ ^15^ ^,^ ^16^ ^,^ ^17^ ^,^ ^18^ ^,^ ^19^ ^,^ ^20^

Previous eating scales, such as the Children’s Eating Behavior Inventory (CEBI)^14^ and the Behavioral Pediatrics Feeding Assessment Scale (BPFAS),^15^ ^,^ ^16^ are used for scientific purposes, although proven to be long for clinical use.^13^ The Montreal Children’s Hospital Feeding Scale (MCH-FS) is applicable through the report of parents or guardians and was designed to identify difficulties with psychometric properties in children from six months to six years and 11 months of age.^12^ Consisted of 14 items, it aims to determine the severity of feeding difficulties, the degree of eating problems, and the level of concern of parents/caregivers.^12^ It is a one-page instrument, freely available and feasible for clinical application, having already been validated in other countries,^13^ ^,^ ^21^ with results similar to the original scale.

Thus, the objective of this study was to carry out the cross-cultural adaptation and validation of the MCH-FS scale into Brazilian Portuguese in children from six months to six years and 11 months of age.

## METHOD

This is a cross-sectional study carried out at *Hospital Materno Infantil Presidente Vargas*. The study was approved by the institution’s Research Ethics Committee, CAAE No. 81513317.0.0000.5329. All parents/caregivers included in the study signed an informed consent authorizing participation in the study.

**Chart 1:**
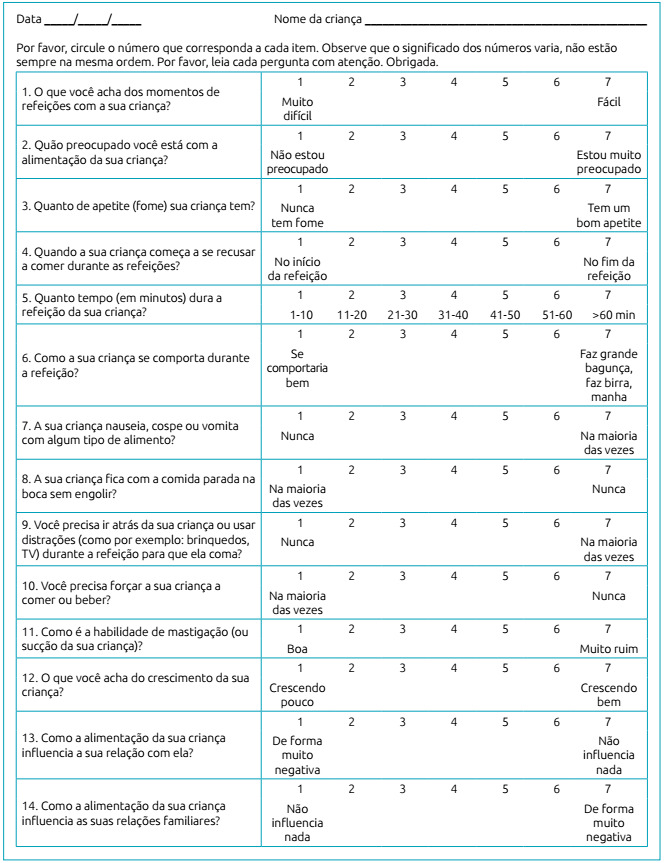
*Escala Brasileira de Alimentação Infantil*.

The scale validation process was carried out according to the methodology previously described in the literature.^22^ ^,^ ^23^ In the initial translation stage, two bilingual translators, having Portuguese as their mother tongue and fluent in English, performed translations of the MCH-FS scale from English to Brazilian Portuguese, in an independent fashion. Brazilian cultural aspects were considered in this process rather than a literal translation. For the synthesis of the translations, the two versions were compared by specialized professionals and each item was evaluated, considering the best way to express it and the influence of cultural aspects. Disagreements related to the questions were adjusted in order to reach a consensus. Then, a single final version of the MCH-FS in Brazilian Portuguese was issued, namely the Brazilian Infant Feeding Scale (*Escala Brasileira de Alimentação Infantil* - EBAI) (Chart 1). The original scale in English and the EBAI were applied to 20 bilingual individuals (main caregivers/feeders of children with typical development) in an interval of 30 days in order to verify the equivalence of the scores between starting with the original scale. Inclusion criteria for bilingual individuals were: to be from the community and to have children with the characteristics of the study sample.

Regarding the minimum sample size for factor analysis, the general rule of ten subjects per variable was used, with a minimum of one hundred subjects in the total sample.^24^ Therefore, a sample size of at least 140 subjects was considered sufficient to perform the analyzes, as the instrument studied has 14 items.

After the scale was translated, agreed to in its final version, back-translated, discussed, and approved by everyone involved in the process, the applicability of EBAI was tested in a sample of 242 parents/caregivers responsible for feeding children, divided into two groups: cases (n=68) and controls (n=174). The 20 bilingual parents were not part of this sample. Recruitment in both groups was carried out consecutively from February to May 2018. The sample included only parents/caregivers of children between six months and six years and 11 months of age. Seven children were excluded from the study, as they presented only dysphagia and were fed by tube or gastrostomy.

The control group was composed of 174 parents/caregivers of healthy children with typical development. Participants were recruited through public recruitment ads in social media. Those interested in participating received informative written material with the objectives of the study, the inclusion criteria, and the explanations about the scale, with the possibility of clarifying doubts with the researchers, if any. Children born at term (>37 weeks) were included in this group, with birth weight ≥2,500 g, with no pre-, peri- or post-natal complications and with adequate neuropsychomotor development, which was assessed through open questions to parents/caregivers. These questions included the fact that children meet the milestones of motor development of the age group.

The authors sent the scale and the instructions for completing it by e-mail to the parents/caregivers who agreed to participate.

The case group consisted of 68 parents/caregivers of children who were undergoing treatment or who had been referred for speech-language assessment due to feeding difficulties in the pediatric inpatient units and in the speech therapy clinic of the hospital, as well as in speech therapists specialized in feeding problems. The scale was filled out in person by the group’s participants after a brief explanation and with the possibility of clarifying doubts throughout the period. This group included children with a diagnosis or suspicion of feeding difficulties, characterized by inability to progress to a texture suitable for their chronological age, refusal of food or acceptance of only small amounts of it, frequent vomiting, resistance struggles with the feeder during meals, prolonged feeding time, use of distractions to increase intake, and using breastfeeding or a bottle at night. These difficulties could be associated or not with dysphagia.

These 68 participants were subdivided into two groups: parents/caregivers of children who had feeding difficulties, but who did not have an associated medical diagnosis or developmental delay (group “feeding difficulty without comorbidities”, n=17) and parents/caregivers of children who had feeding difficulties and an associated medical diagnosis, such as prematurity and gastrointestinal, cardiorespiratory, genetic, and neurological disorders (group “feeding difficulty with comorbidities”, n=51). Neuropsychomotor development assessment was carried out through the same open questions asked to ­parents/­caregivers in the control group. Children who did not meet the expected motor development milestones for the age group were considered delayed.

To verify the reliability of the test/retest of the study, 25 parents/caregivers from the case group, selected at random, filled out the scale again, 10 to 15 days from the first application. The selection was carried out using the WINPEPI 11.65 software, using a list of random numbers among the ones corresponding to the parents/caregivers of this group. The 25 selected subjects received the questionnaire by e-mail to retest. The responses submitted in both moments were compared to one another.

Quantitative variables and ordinal variables were described by the mean (standard deviation - SD) and the median (interquartile range). The Kolmogorov-Smirnov test was performed to test the normality of the data. Items in the three groups were compared by the Kruskal-Wallis test or by analysis of variance, and between two groups by the Mann-Whitney test. Categorical variables were described by frequencies and percentages and compared using the χ^2^ test. Quantitative variables were correlated by Spearman’s correlation coefficient. To assess the internal consistency of the dimensions, Cronbach’s alpha was used. Test/retest results were compared using Pearson’s correlation coefficient and, later, their means, using the Student’s *t* test for paired samples. Performance of the raw score were calculated for the suggested cut-off point of 45: sensitivity, specificity, positive predictive value, negative predictive value, and accuracy. A Receiver Operating Characteristics Curve (ROC curve) was performed to assess the ability of the raw score to discriminate between cases and controls. A significance level of 5% was considered statistically significant. Data analysis was performed using the Statistical Package for the Social Sciences (SPSS), version 20.0 (IBM SPSS^®^ Statistics, São Paulo, Brazil).

## RESULTS

In this study, data were collected from 242 participants, 174 (71.9%) of the control group and 68 (28.1%) of the case group. In the latter, 17 (7.0%) belonged to the subgroup “feeding difficulty without comorbidities” and 51 (21.1%), to the subgroup “feeding difficulty with comorbidities”. Table 1 shows the characteristics of both case and control groups. With regard to bilingual parents, although score values were not identical for all participants, the ratings obtained in in both languages were exactly the same.

**Table 1:** Characteristics of the studied groups.

	Controls (n=174)	Feeding difficulty without comorbidities (n=17)	Feeding difficulty with comorbidities (n=51)	p-value
Age of the mother *	36.5 (33.8-39.0)^a^	34.0 (28.5-36.5)^a,b^	35.0 (29.0-38.0)^b^	0.008*
Education of the mother**				
Incomplete Elementary School	-	-	1 (2.0)	<0.001**
Elementary School	9 (5.2)	1 (5.9)	11 (21.6)	
High School	37 (21.3)	5 (29.4)	18 (35.6 )	
Higher Education	128 (73.6)	11 (64.7)	21 (41.2)	
Age of the father*	38.0 (35.0-41.0)	35.0 (30.0-37.0)	36.0 (31.0-41.0)	0.032*
Education of the father**				
Incomplete Elementary School	2 (1.2)	-^a,b^	4 (8.3)	<0.001**
Elementary School	8 (4.6)	3 (17.6)	10 (20.8)	
High School	47 (27.2)	5 (29.4)	15 (31.2)	
Higher Education	116 (67.1)	9 (52.9)	19 (39.6)	
Age (months)*	37 (24-56)	18 (13-33)	27 (15-40)	<0.001*
Gestational age*	39 (38-40)	39 (38-40)	37 (33-39)	<0.001*
Weight at birth (g)*	3,255 (2,984-3,535)	3,150 (2,655-3,699)	2,730 (1,795-3,095)	<0.001*
Male**	84 (48.3)	8 (47.1)	30 (58.8)	0.399**
Firstborn**	117 (67.2)	9 (52.9)	23 (45.1)	0.013**

Different letters represent different groups; *values represented by median (25th percentile - 75th percentile) and compared using the Kruskal-Wallis test; **values in number (%) and compared using the χ^2^ test.

When comparing the scores of each of the 14 questions on the scale, there was no significant difference between the subgroups with and without comorbidities, except for question 1, with lower scores for those with comorbidities (p=0.025), and questions 5 and 12, with higher scores for those with comorbidities (p=0.018 and p=0.046, respectively). The overall raw scores were not statistically different between the subgroups with and without comorbidities, which allowed for further analysis with the two groups together.

When comparing the scores of each of the 14 questions between the control and the case groups, it presented significantly higher values for all items, except for item 5, as well as higher raw scores and T-scores (Table 2). The difference in final scores in the case and the control groups were statistically significant (p <0.001), with a higher frequency of children with values considered high (79.4%) in the group of cases.

**Table 2:** Comparison of descriptive measures (mean, median, and interquartile range) of the scores of the *Escala Brasileira de Alimentação Infantil* between cases and controls.

Items	Cases (n=68)					Controls (n=174)					p-value*
	Mean	Median	Standard deviation	Percentage		Mean	Median	Standard deviation	Percentage		
				25	75				25	75	
1	4.47	5.00	2.00	3.00	6.00	2.61	2.00	1.55	1.00	3.25	<0.001
2	5.47	6.00	1.77	4.00	7.00	3.68	3.00	2.05	2.00	5.00	<0.001
3	3.69	4.00	2.06	1.00	5.00	2.22	1.50	1.55	1.00	3.00	<0.001
4	4.63	5.00	2.15	3.00	6.75	2.82	2.00	2.13	1.00	4.00	<0.001
5	3.00	3.00	1.40	2.00	4.00	2.66	2.50	1.05	2.00	3.00	0.088
6	4.06	4.00	1.88	3.00	5.00	2.83	2.00	1.76	1.00	4.00	<0.001
7	4.13	4.00	2.11	2.00	6.00	1.85	1.00	1.31	1.00	2.00	<0.001
8	3.37	3.00	2.05	1.00	5.00	1.70	1.00	1.32	1.00	2.00	<0.001
9	4.21	5.00	2.45	2.00	7.00	3.01	2.00	2.15	1.00	4.25	0.001
10	3.54	3.00	2.02	2.00	5.00	2.23	2.00	1.66	1.00	3.00	<0.001
11	3.49	3.00	2.18	1.00	5.00	1.33	1.00	1.03	1.00	1.00	<0.001
12	3.21	3.00	2.20	1.00	4.75	1.53	1.00	1.18	1.00	2.00	<0.001
13	3.18	3.00	1.90	1.00	4.00	1.99	2.00	1.27	1.00	3.00	<0.001
14	3.09	3.00	1.98	1.00	4.00	1.89	1.00	1.22	1.00	2.00	<0.001
Total raw score	53.53	53.00	14.75	46.00	64.00	32.36	30.50	11.28	23.00	39.00	<0.001
*T-score*	66.53	66.00	11.64	61.00	75.00	49.89	48.50	8.84	43.00	55.00	<0.001

* Data compared using the Mann-Whitney test.

There was a difference between the case group and the control group regarding the severity of the feeding disorders. In the former, problems with mild difficulty were reported in 27.9% of cases, moderate difficulty in 17.6%, and severe in 33.8%, while, most reported no eating difficulties (86.8%) in the latter.

Table 3 describes the comparison between cases and controls stratified by age range. It is possible to observe that both in the age group of six to 24 months and in the age group from 25 to 83 months, the group of cases had significantly higher values than the control group (p<0.001). There was a statistically significant difference between the groups (p <0.001) in the analysis of covariance (ANCOVA), adjusted for comparison between the T-score groups aged ≤24 months and > 24 months.

**Table 3:** Comparison between cases and controls stratified by age group.

Age in months	Cases			Controls			p-value
	n	mean±SD	median (IR)	n	mean±SD	median (IR)	
6-24	35	65.1±12.6	65.0 (61.0-75.0)	48	49.6±6.8	50.0 (44.0-54.0)	<0.001
25-83	33	68.0±10.6	68.0 (62.0-75.5)	126	50.0±9.5	48.0 (43.0-55.3)	<0.001

SD: standard deviation; IR: interquartile range; data compared between groups using the Mann-Whitney test.

Good internal consistency was found, in both groups, for all items on the scale (Cronbach’s alpha=0.79). Using the suggested cutoff point of 45, the raw score differentiated control cases with sensitivity of 79.4%, specificity of 86.8%, positive predictive value of 70.1%, negative predictive value of 91.5%, and accuracy of 84.7%.

**Figure 1:**
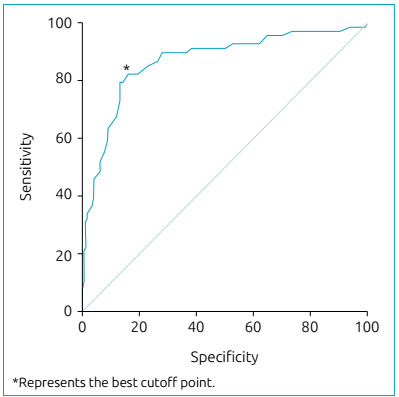
Receiver Operating Characteristics Curve of the raw score to discriminate cases and controls. Best sensitivity/specificity ratio was obtained with a cut-off point of 42.5, with a sensitivity of 82.4% and specificity of 83.9%.

The area under the ROC curve was 0.87 (p<0.001; 95% confidence interval [95%CI] 0.81-0.92) (Figure 1). As shown in Figure 1, the best sensitivity/specificity ratio was obtained with the cutoff point of 42.5, with a sensitivity of 82.4% and specificity of 83.9%.

The correlation of the total score between test and retest was considered strong (r=0.92; p<0.001). The mean (SD) of the score was 63.2 (10.0) on the test and 62.7 (11.1) on the retest, with no statistically significant difference between the two moments (p=0.264).

## DISCUSSION

The results of this study demonstrated that the EBAI may be applicable in Brazil. It is, therefore, a useful tool for the identification of feeding difficulties in children from six months to six years and 11 months of age within the Brazilian cultural context.

The translation and cross-cultural adaptation processes that resulted in the development of the EBAI made it possible to adapt the original scale and make it useful for use in Brazilian culture. To this end, it was taken into account that, in the process of cross-cultural adaptation, it is crucial to maintain the coherence of the concepts and properties apprehended by the original version.^22^ In addition, each item of the initial protocol must be adapted in order to achieve semantic, linguistic, and contextual equivalence between the original and the adapted versions, so that it can also retain its equivalence in a specific situation of use.^23^

The score obtained by the control group for question 2 (“How concerned are you with your child’s feeding?”) was higher when compared to the control groups in other countries where the scale has already been validated.^12^ ^,^ ^13^ ^,^ ^21^ According to previous studies,^25^ ^,^ ^26^ it is known that the feeding practices of Brazilian mothers are loaded with symbolic values ​​and heavily involved in cultural aspects. In this cultural relationship, when children do not eat or reject food, they are “disqualifying” their mother’s competence to ensure adequate nutrition.^25^ In addition, Brazilian mothers reported concerns on the amount of food their children ingested; therefore, such mothers seemed to value good feeding rather based on the amount of food that children were able to ingest than on the energetic density that food provided.^25^ Brazilian mothers also believe that eating well equals eating a lot.^26^ Thus, it is believed that the highest score found by the controls in question 2 suggests that the greatest concern may be a Brazilian cultural issue. However, this data did not affect the total score of the present study when compared to the original version.^12^

There was a statistically significant difference between the control group and the case group in relation to all questions, with the exception of question 5. This result demonstrates that the questions that make up the instrument are efficient in differentiating children with or without feeding difficulties. This difference in relation to the shorter observation of feeding time seems to be a cultural issue rather than a translation problem, since the total mean score found in this study is similar to the data already described in the literature.^12^ ^-^ ^13^ ^,^ ^21^ These results highlight the universality of feeding parameters regardless of culture, whether in North America, Europe, Southeast Asia or, now, South America.

In the comparison by age group (≤24 months and >24 months), the cases obtained a significantly higher mean than the controls. However, in the group of cases, older children had higher mean scores than younger ones, which suggests that there is no decrease in feeding difficulties with increasing age. This difference demonstrates a tendency to maintain feeding difficulties and has been previously described.^8^ ^,^ ^13^ A possible explanation for this finding can be based on the likely increase in stress levels and other negative interactions between caregivers and children during meals.^8^ ^,^ ^13^ Caregiver-child interactions are often mentioned as a contributing or sustaining factor for the persistence of feeding difficulties.^8^

Higher means in the group of cases from six months of age are in accordance with those described in other studies reporting the early onset of feeding difficulties.^27^ ^,^ ^28^ According to these reports, the onset of such difficulties occurs before the first year of life in 50% of children,^27^ and at 18 months of age or earlier in up to 75% of children.^28^ The findings in the present study regarding the persistence of feeding difficulties with advancing age, associated with the onset of symptoms before 25 months of age, further reinforces the need for validation of a screening instrument that can be used for babies from six months of age in order to identify feeding difficulties before the establishment of avoidant behavioral patterns.^3^ To date, two instruments were published for that age group: the MCH-FS^12^ and the Pediatric Eating Assessment Tool (PediEAT),^3^ ^,^ ^18^ ^,^ ^20^ both not adapted and cross-culturally validated for Brazilian Portuguese. The EBAI, the Brazilian version of the MCH-FS developed in the present study, has an internal consistency similar to the original version^12^ and the Dutch and Thai versions,^13^ ^,^ ^21^ demonstrating good sensitivity and specificity with the suggested cutoff point of 45. These findings are also in agreement with those of the original scale.^12^ The area under the ROC curve in the present study suggests good accuracy for the raw score between cases and controls, being quite close to the original scale.

With regard to the difference found in the demographic data of parents/caregivers, the ones in the control group were older and had higher levels of education than those in the subgroup “food difficulties with comorbidities”, which shows a higher socio-cultural level in the control group. Such data may represent a limitation in the study, since parents/caregivers with greater access to information can interpret the questions differently. It should also be considered that there is no other validated Brazilian instrument to compare these findings.

The EBAI’s proposal for cross-cultural adaptation and validation proved to be reliable, reaching initially set objectives. Results showed that the questionnaire can be understood by the target audience, being able to achieve the objectives described in the original scale, and has appropriate psychometric measures for the identification of feeding disorders in Brazilian children from six months to six years and 11 months of age. Future use of the instrument in control and case groups with a similar sociodemographic profile may reduce the potential biases of the present study.

It is concluded that the availability of EBAI will allow health professionals to use a reliable and cost-free tool for the rapid detection of dietary problems, contributing to the early identification of these problems and the consequent faster referral to specialized treatment. Thus, it is expected to minimize the damage resulting from organic, social, financial, and emotional stress that feeding problems bring upon children and their families.
